# An overview of pim kinase as a target in multiple myeloma

**DOI:** 10.1002/cam4.5797

**Published:** 2023-05-10

**Authors:** Zhaoyun Liu, Yunhe Zhang, Yixuan Guo, Hao Wang, Rong Fu

**Affiliations:** ^1^ Department of Hematology Tianjin Medical University General Hospital Tianjin People's Republic of China

**Keywords:** bone disease, immune, multiple myeloma, pim kinase, tumor load

## Abstract

Multiple myeloma (MM) is the second common hematologic malignancy manifesting as a clonal proliferation of plasma cells in the bone marrow. In recent years, high expression and activity of pim kinase have been found to be associated with both the progression and prognosis of a significant proportion of malignant diseases. Therefore, pim kinase has become a potential therapeutic target in the treatment of MM and some pim kinase inhibitors have demonstrated good efficacy in clinical trials. Based on nearly the entire literature searched from PubMed in the field of pim kinase in MM, the paper concluded how pim kinase got involved in the proliferation of myeloma cells, the progression of bone disease infiltration, and even in the regulation of the immune microenvironment. Next as a very promising drug, the effectiveness of pim kinase inhibitors as single agents or in combination with other drugs in the treatment of MM was also summarized. Our analysis will guide the clinical use of pim kinase inhibitors for managing tumor load and bone disease in MM patients.

## Graphical abstract

In multiple myeloma, pim kinase inhibitors inhibit tumor growth and reduce bone destruction while modulating the immune microenvironment. Such a promising drug requires more clinical trials and basic research to provide a more comprehensive treatment option for myeloma.

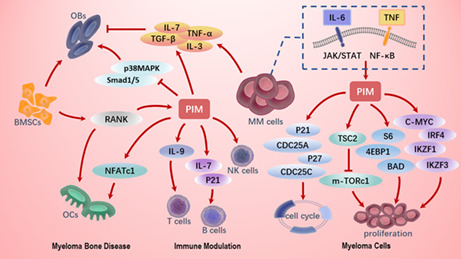

## 1 INTRODUCTION

Multiple myeloma (MM) is an incurable malignant disease of the hematologic system from plasma cells. Tumor load, bone disease, and immunity are the three fundamental issues that have drawn clinical attention in MM. The family of pim kinases comprises three isozymes: pim‐1, pim‐2, and pim‐3, all of which are reported to be overexpressed in several hematologic malignancies.^1^ Several pathways and molecules involved in tumor progression, such as the well‐known interleukin (IL)‐6/STAT3 and TNF/NF‐κB pathways, have recently been found to be involved in the transcription of pim kinases as well.^2^ Targeting these pathways would inhibit myeloma cell proliferation by promoting cell cycle arrest and activating metabolic pathways, such as glycolysis and lipid biosynthesis.^3^ For bone disease, the use of a pim kinase inhibitor considerably reduced myeloma‐induced bone destruction in a mouse model. The decrease in osteoclastogenesis and bone resorption, as well as the increase in osteoblast (OB) activity and mineralization, could be a reasonable cause.^4^ The immunomodulation of pim kinase has been recently reported; however, the interaction between the immune microenvironment and pim kinase in MM cells has been studied less. The present review focuses on the interaction between pim kinase and MM, the mechanism underlying the contribution of pim kinase to myeloma progression and bone loss, and efficacy of pim kinase inhibitors in patients with MM.

## 2 BACKGROUND

First identified in 1986, pim‐1 is a putative oncogene that is activated in murine leukemia virus‐induced T‐cell lymphomas as a result of provirus insertion.^5^ Transgenic mice carrying the pim‐1 gene are able to complement the upstream immunoglobulin enhancer and the downstream murine leukemia virus long terminal repeats.^6^ The human‐derived pim‐1 gene, which shares 53% of the nucleotide sequence with the mouse‐derived pim‐1 gene^7^, ^8^ can be isolated from the K562 library and sequenced,^9^ involving six exons and five introns as well as multiple GC boxes as promoters (CCGCCC). Mature pim‐1 mRNA is approximately 3 kb in length and can translate a protein containing 313 amino acids without a transmembrane zone.^10^, ^11^ The 33‐kDa pim‐1 product is a protein serine kinase with autophosphorylation activity.^12^, ^13^ It is overexpressed in 30% of cell samples from patients with hematologic malignancies, particularly myeloid and lymphoid leukemias.^14^ In some myeloid cells, except neutrophils and monocytes, GM‐CSF and IL‐3 can induce pim‐1 production, suggesting that pim‐1 is an important intermediate in some cytokine‐induced transmembrane signals or responses in myeloid leukemia.^15^, ^16^, ^17^ Fission yeast mutant down‐dialing containing a pim‐1 mutation can undergo mitotic chromosome condensation and spindle formation without completion of S phase and in the absence of the CDC25 mitotic inducer.^18^ In 1993, a mouse model of pim‐1 gene deficiency was established, and it was also found that pim‐1 deficiency leads to erythrocyte microcytosis.^19^ Crossing Emu‐pim‐1 gene mice with Fas gene‐deficient mice leads to a massive proliferation of lymphocytes in the daughter mouse generation and protects against dexamethasone‐induced apoptosis.^20^ Pim‐2 was first reported in 1995. When studying the cooperation between pim‐1 and the proto‐oncogene myc, a PCR‐based screen identified a close homologous gene pim‐2, X‐linked and sharing 53% amino acid homologous sequence with pim‐1.^21^ Pre‐B‐cell leukemia appears in neonate Emu‐myc with pim‐2 in bi‐transgenic animals.^22^ The human pim‐2 gene is 90% identical to the murine pim‐2 gene at the structural level. Its 2.2 kb transcript can be expressed in hematopoietic tissues leukemia and lymphoma cell lines (K‐562, HL‐60, and RAJI) are highly expressed.^23^ The pim‐3 gene was initially identified in rat pheochromocytoma cells in 1998 as a depolarization‐inducing gene, 76% identical to pim‐1,^24^ and had also been obtained by homology screening techniques.^25^ Pim‐3 is aberrantly expressed in human HCC tissues and hepatocellular carcinoma cell lines while being involved in the proliferation of human hepatocellular carcinoma cell lines.^26^ Pim‐3 inactivates Bad phosphorylation and maintains Bcl‐X(L) expression, thereby preventing apoptosis in all human pancreatic cancer cells.^27^

## 3 PIM KINASE IN MM

**FIGURE 1:**
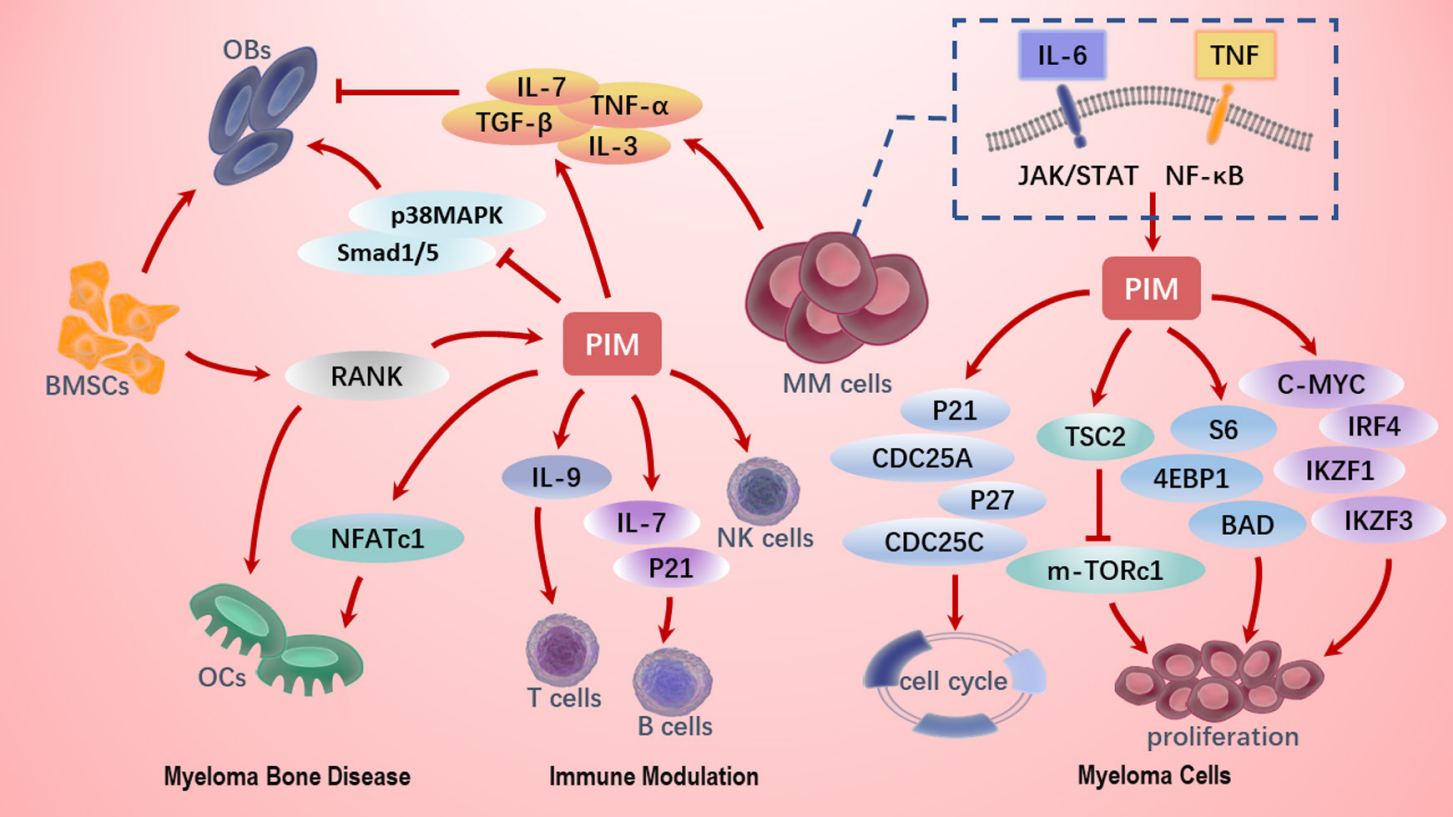
Targeting pim kinase in multiple myeloma.

Pim‐1 is overexpressed in hematologic diseases and solid tumors, pim‐2 in myeloma, lymphoma, and leukemia, and pim‐3 in substantial organ tumors.^28^ In 41 newly diagnosed patients with MM, substantially higher levels of pim‐2 than the other two were observed in CD138^+^ myeloma cells. pim‐1 levels were also higher than pim‐3 levels.^29^ (Figure S1) In addition, bone marrow stromal cells (BMSCs) can augment pim expression in MM cells.^30^ Pim‐1 activates endonucleases responsible for DNA breaking during apoptosis and inhibits DNA repair systems to promote inter‐ribosomal breaks in chromosomal DNA in several cell lines derived from mouse NS‐1 myeloma cells.^31^ MiR33b significantly inhibits pim‐1 at the gene and protein levels in MM1.s cells, but miR33b no longer mediates apoptosis in cells overexpressing pim‐1 (Figure 1).^32^

### 3.1 Regulation of MM cell cycle

The cell cycle is the process by which cells reproduce.^33^ The cell cycle includes the interphase (G1, S, and G2) and mitosis (M).^34^ G0 phase can exist before the M phase, indicating that the cell will leave the cell cycle and enter a quiescent state.^35^ Pim kinase acts in the downstream effector functions, especially as a promoter of G1/S phase progression during the cell cycle.^36^ Pim kinase is involved in the regulation of the MM cell cycle by acting on p21Cip1/Waf1(CDKN1A), p27Kip1(CDKN1B), and CDC25A/C via their phosphorylation pathways,^37^ but there is no consensus on whether pim kinase accelerates the cell cycle or causes it arresting.

Pim‐1 phosphorylates p21Cip1/Waf1 at Thr‐145, exerting an inhibitory effect on the G1/S phase^28^ and increasing the proportion of sub‐G1 cells in RPMI8226 cells.^38^ Pim‐1 enhances CDC25A activity and inhibits CDC25C activity by phosphorylating them,^28^ which however reduces the CDC2‐cyclin B1 complex, an essential driver of the G2/M phase transition, leading to cell cycle arrest in MM1s cells.^39^ Another voice is that pim kinase speeds up the cell cycle. P21 is upregulated after downregulation of pim‐2 using short interfering RNA, which, in turn, causes G0/G1 cell cycle arrest in RPMI‐8226 MM cells.^40^ Pim kinase phosphorylates p27Kip1 at Thr‐157 and Thr‐198.^41^ In contrast, phosphorylated p27Kip1 could not mediate G2/M phase arrest in MM.1S cells.^42^ LT‐171‐861, a multiple kinase inhibitor that inhibits pim kinase, decreases CDK1 expression and increases CDK1 phosphorylation, inducing G2/M arrest in U266 and RPMI8226 cells.^43^ Moreover, pim447 blocks the myeloma cell cycle at the G1‐to‐S transition by downregulating cyclin D2 and E1, which may be owing to the reduction of c‐Myc levels.^29^

### 3.2 Proliferation and anti‐apoptotic activity of MM cells

Pim, acting as an oncogene, causes differences in a variety of tumors.^44^ The expression of pim kinase is involved in some signaling pathways, such as JAK/STAT3, NF‐κB, and PI3K‐AKT, that affect the activity of many downstream molecules by phosphorylation, such as BAD, MYC, and mTOR‐C1. The complex mechanism of pim kinase in MM cell proliferation and anti‐apoptotic activity is systematically described below.

Pim‐1 can increase the stability and transcriptional activity of c‐Myc via its phosphorylation at serine 62 (S62).^45^, ^46^ However, the inhibition of phosphorylation by compound 7594‐0037 (a novel c‐Myc inhibitor) leads to a high mortality rate in RPMI‐8226 and U266 cells.^47^ Pim‐1 has also been reported to directly target miR‐33a‐5p. High levels of miR‐33a‐5p and low levels of pim‐1 blocked the promotion of apoptosis in MM cells.^48^

Based on the current data, pim‐2 was expressed at a higher level in myeloma cells than pim‐1 and pim‐3, suggesting a more important role for pim‐2 in MM biology.^49^ Pim‐2 promotes MM cell survival by activating the JAK2/STAT3 pathway via IL‐6^50^ and the NF‐κB pathway via the TNF family cytokines.^51^ Pim‐2 directly phosphorylates TSC2, a negative regulator of mTOR‐C1, on Ser‐1798 and drives MM cell proliferation.^52^ LT‐171‐861, which can inhibit multiple kinases, including pim‐2, suppressed the proliferation and mediated the death of MM cells. A decline in phosphorylation products, such as 4EBP1 and BAD, was also observed, suggesting a correlation with the above process.^43^ Pim‐2 has shown to inhibit the activation of the DNA damage response (DDR) pathway through ATR regulation. Knockdown of pim‐2 upregulates downstream DDR markers in MM cells.^53^ However, the upregulation of pim‐2 is independent of the PI3K/Akt pathway, suggesting that pim‐2 inhibitors, in combination with PI3K/Akt inhibitors, contribute to anti‐MM efficacy.^30^ It was also discovered that sustained downregulation of IKZF1, IKZF3, and their downstream molecules such as c‐Myc and IRF4, by inhibiting pim kinase via SGI1776 or CX5268, caused very low survival rates of MM1R and MM1S.^54^

Pim‐3 has been noted in adenocarcinomas^28^ and has been associated with colorectal^55^, ^56^ and gastric cancers.^57^ However, its expression in MM is rarely reported, and more studies are required to demonstrate its role in myeloma cells.

## 4 PIM KINASE IN MM‐RELATED BONE DISEASE

MM is highly likely to cause bone diseases.^58^ The rate of patients with osteolytic lesions when first diagnosed with myeloma is up to 80%. They experience bone‐related events such as pathological fractures, osteoporosis syndrome, and compression of the spinal cord.^59^ Myeloma cells not only create a cellular microenvironment to guard MM cells against apoptosis^51^ but also stimulate RANK ligand‐mediated osteoclastogenesis and suppress osteoblastic differentiation, which leads to extensive bone destruction by producing a variety of cytokines.^60^

### 4.1 Pim kinase and osteoclasts

RANKL is capable of inducing pim‐1 expression and calcium oscillations, the latter of which is required for NFATc1 induction and osteoclastogenesis.^61^ RANKL can activate kinase 1 (TAK1) and NF‐κB by inducing TGF‐β. However, pim‐1 overexpression blocked these processes and affected NFATc1 expression during osteoclastogenesis.^62^ In co‐cultures of bone marrow cells with MM cells, inhibition of TAK1 attenuated RANKL‐enhanced osteoclastogenesis and restored the suppression of osteoclastogenesis overproduced in MM cells.^54^ Pim‐1 also directly regulates the transcriptional activity of NFATc1, an OB‐specific gene, and subsequently induces the expression of OB‐associated receptors. However, the same experiments on the other two kinases did not reveal similar findings to pim‐1.^62^

Osteoclasts (OCs) upregulated Pim‐2 expression in MM cells mainly through the IL‐6/STAT3 and NF‐κB pathways, respectively. Pim‐2 short interfering RNA reduced the viability of MM cells when co‐cultured with BMSCs or OCs.^51^ Pim‐2 was expressed only in proteinase K‐positive OBs. Thus, treatment with a pim‐2 inhibitor (SMI‐16a) reduced the number of proteinase K‐positive OBs in an animal model of MM. In vitro, the addition of SMI‐16a abolished RANKL‐induced [Ca^2+^]i oscillations.^63^ NFATc, a critical transcription factor for osteoclastogenesis, automatically amplifies RANKL‐triggered intracellular Ca^2+^ ([Ca^2+^]i) oscillations.^64^ This suggests that pim‐2 affects the [Ca^2+^]i oscillations of RANKL and upregulates NFATc1.^63^

Calcium matrix‐coated slides were used to observe resorptive pits after pim447 treatment. A dose‐dependent reduction was detected. Pim447 reduced not only NFATc1, but also the level of a protease (cathepsin K) responsible for the degradation of the organic bone matrix. Other molecules involved in matrix resorption, such as MMP9 and ATP6V1A, were also substantially reduced following therapy, suggesting a role for pan‐pim kinase inhibitors in preventing bone resorption.^29^

### 4.2 Pim kinase and OBs

Pim‐2 kinase is also known as a common downstream molecule for the inhibition of osteoblastogenesis in myeloma^60^ and is upregulated in BMSC by major inhibitors of bone formation overproduced in MM, such as IL‐3, TGF‐β, TNF‐α, and IL‐7. Among these, TGF‐β strongly inhibits terminal OBs differentiation.^65^ Suppression of osteoblastogenesis by all the above inhibitory factors can be restored by pim‐2 short‐interfering RNA together with SMI‐16a.^66^ BMP‐2 (Bone morphogenetic protein‐2) phosphorylates Smad1/5 and p38MAPK^67^ and can induce directed differentiation and proliferation of undifferentiated BMSCs into chondrocytes and OBs.^68^ Overexpression of pim‐2 disrupted the phosphorylation of both molecules in MC3T3‐E1 cells.^66^

## 5 CLINICAL EFFICACY OF PIM KINASE INHIBITORS IN MM

Transgenic mouse models and overexpression studies in cell lines have confirmed that pim kinase is involved in the transduction of many cellular signaling pathways, thereby promoting tumorigenesis, especially in blood tissues. This supports the hypothesis that pim kinase inhibitors are small molecular inhibitors for MM patient therapy.^2^, ^69^ Newly discovered pan‐pim kinase inhibitors, such as pim447, are presently entering clinical trials that have demonstrated positive effects in MM. Here, we summarize the current data of all reported pim kinase inhibitors with promising single‐agent activity and combination therapy in MM.

### 5.1 Single‐agent therapy in MM

#### 5.1.1 ATP‐competitive pim kinase inhibitors

Pim447 (LGH447) is an oral, potent pim kinase inhibitor that has entered clinical trials according to a study first performed in MM patients (NCT01456689) (Table 1).^70^, ^71^ A preclinical study showed that pim447 is active against pim‐2‐dependent MM cells and tumor enlargement in a mouse subcutaneous tumor model^52^ by suppressing mTOR‐C1 signaling via TSC2 and inhibiting phosphorylation of the Bcl‐2‐associated death promoter.^34^ However, low doses of pim447 (0.1–1 μM) mediated cell death primarily through cell cycle arrest rather than induction of apoptosis, which is concurrent with clinical information, as many patients were stabilized after monotherapy with pim447.^29^ Moreover, the bone tumor load was substantially reduced in an orthotopic human xenograft model of MM.^52^

**TABLE 1:** Mechanism of the newly discovered pim kinase inhibitors in myeloma cells.

Drug	Molecular structure	Mechanism	Object	References
SGI‐1776	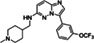	Induces lipidation of LC3I to LC3II, promoting cellular autophagy, without affecting cellular metabolism	U266 cells	51, 72, 73
			MM1.s cells	
LGB321	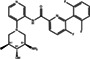	Significantly inhibits the phosphorylation of mTOR‐C1 downstream molecules p70S6K, S6RP, 4EBP1, and eIF4G, thereby suppressing proliferation	KMS‐11.luc cells	^52^
			KM‐26 cells	
			MM1.s cells	
			RPMI‐8226 cells	
LGH447	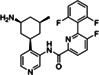	Inhibits mTOR‐C1, decreases phosphorylation of 4EBP1 and S6P, inhibits BAD phosphorylation and cleavage of PARP and cystein 3, 7, 8, 9, and promotes apoptosis	MM1.s cells	29, 34, 52, 70, 71
			RPMI‐8226 cells	
JP11646	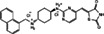	Inhibits phosphorylation of 4EBP1 and BAD promoting apoptosis	MM1.s cells	^74^
			U266 cells	
SMI‐16a	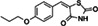	Reduces MCL‐1 and inhibit proliferation under weakly acidic conditions	RPMI‐8226 cells	63, 66, 75
			U266 cells	
INCB053914	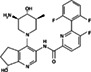	Inhibits phosphorylation of p70S6K, 4EBP1, and BAD promoting apoptosis	KMS‐12‐BM cells	^76^
Compound 9c	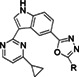	Inhibits phosphorylation of BAD promoting apoptosis	KMS‐12‐BM cells	^77^
Compound 29	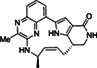	Inhibits phosphorylation of BAD promoting apoptosis	KMS‐12‐BM cells	^78^

According to the clinical trial of pim447 in relapsed/refractory MM (NCT01456689), 77 Caucasian patients were included. The overall response rate (ORR) was 8.9%, with disease control and clinical benefit rates of 72.2% and 25.3%, respectively. A dose intake of 200 mg resulted in one patient (1.3%) achieving the desired PR and 10.9 months as median progression‐free survival.^70^ A study conducted to determine the difference between the maximum tolerated dose (MTD = 300 mg) and recommended expanded dose (250 mg) of pim447 in Japanese relapsed/refractory MM patients (NCT02160951) revealed that among all the patients, the ORR was 15.4% with a disease control rate and clinical benefit of 69.2% and 23.1%, respectively. The most common adverse event related to pim447 was thrombocytopenia, which accounted for 76.9% of the patients with both anemia and leukopenia (53.8%).^37^, ^71^

SGI‐1776 and AZD1208 are potent ATP‐competitive inhibitors and are in phase 1 clinical trials for the treatment of refractory prostate cancer, RR NHL patients,^72^, ^79^ and recurrent or refractory acute myeloid leukemia (AML) patients,^73^ respectively. SGI‐1776 therapy contributes to apoptosis in myeloma patients with primary CD138^+^ cells <10%; however, a 70% reduction in DNA synthesis was observed at 3 μM in U266 cells. Autophagy was induced in all types of cells (25%–70% in U266 cells, 8%–52% in MM.1S, and 19%–21% in CD138^+^ cells).^51^ However, experimental data on AZD1208 in myeloma cell lines are not yet available.

INCB053914, another selective ATP‐competitive inhibitor of pim kinase, inhibited pro‐apoptotic protein BAD phosphorylation in KMS‐12‐BM cells (IC50 = 134 nM), which is similar to mice bearing MM tumors (IC50 = 145 nM). Growth suppression of 43%, 71%, 77%, and 88% of KMS‐12‐BM tumor was observed with doses of 25, 50, 75, and 100 mg/kg BID, respectively.^76^

LGB321 is a small‐molecule selective ATP‐competitive pim kinase inhibitor. MM cells were extensively sensitive to LGB321, of which 14 MM cell lines had a GI50 lower than 1 mmol/L compared to that of NHL, CML, ALL, and AML cell lines. Tumor stasis got the maximal in vivo effect in the KMS‐111 luciferase xenograft model (100 mg/kg QD).^80^

SMI‐16a and SMI‐4a are compounds from the thiazolidine‐2,4‐dione‐family, that can reduce the pim‐2 protein activity without affecting its mRNA. Acidic (pH = 6.8) enhanced anti‐MM actions and decreased pim‐2 protein levels in MM cells, but AZD1208 and pim447 did not have similar results. SMI‐16a also restores Dox's anti‐MM effects in acidic conditions.^66^, ^75^

#### 5.1.2 Non‐ATP competitive pim kinase inhibitor

JP11646, a non‐ATP competitive inhibitor with selectivity for pim‐2, shows great anti‐proliferative effects, especially in MF‐signature cell lines (MM1.S) with a GI50 of 5 nM. It inhibits MM proliferation and has viability 4–760 times greater than other ATP‐competitive pim inhibitors. JP11646 progressively decreased pBAD (S112) and p4EBP1 (S65) with time in MM1.S and U266 cells and dose dependently minimized phosphorylation of the anti‐apoptotic factor MCL1 at Ser159 or Thr163 to 10% in MM1.S and 30% in U266 cells, because of its pim‐2 inhibitory effect.^74^

#### 5.1.3 Other newly discovered pim kinase inhibitors

A class of quinoxaline‐pyrrolodihydropiperidinones, pim‐1, and pim‐2 kinase inhibitors, exemplified by 9C, exhibited double‐digit nanomolar activity and potently reduced the viability of KMS‐12‐BM cells (IC50 = 151 nM). In the KMS‐12‐BM tumor mouse model, orally administered 9C at doses of 10–100 mg/kg resulted in a remarkable decline in tumors. Furthermore, the dose of 100 mg/kg completely silted tumor growth.^77^

A potent and selective series of quinazolinone–pyrrolopyrrolone compounds have been discovered. Compound 1 is efficient at the molecular (IC50 = 0.3 nM for pim‐1 and pim‐2) and cellular level (IC50 = 23 nM in KMS‐12 BM), and is also an efficient therapeutic in the myeloma model of mice from KMS‐12 BM with 78% TGI at 50 mg/kg QD.^81^ Compound 28 demonstrated the targeting activity of pim in an in vivo pharmacodynamic assay, with significant inhibition of BAD phosphorylation in KMS‐12‐BM cells. In a 2‐week mouse xenograft model, daily administration of Compound 28 at 100 mg/kg resulted in 33% tumor regression.^82^

Compound 22 m, a sub‐nanomolar inhibitor of the pim‐1 and pim‐2 isoforms (IC50 values of 0.024 nM and 0.095 nM, respectively), effectively inhibited the phosphorylation of BAD in KMS‐12‐BM, a cell line that expresses high levels of all pim isoforms, to regulate myeloma cell proliferation and apoptosis.^83^

### 5.2 Drugs combined with pim kinase therapy in MM

Recently, several new drugs for MMs, such as immunomodulatory drugs (IMiDs), proteasome inhibitors (PIs), monoclonal antibodies, as well as several other small molecule inhibitors, have emerged.^69^, ^84^ Several treatment options for relapsed and refractory MM have been used in clinical applications.^85^ As pim kinase has similar downstream products to other oncogenic kinases and has totally different but reciprocally compensating effects in different tumors,^86^ the rational combination of pim kinase inhibitors with anticancer drugs targeting other signaling pathways for cell proliferation and survival has attracted increasing interest (Table 2).

**TABLE 2:** Combination drug therapy in multiple myeloma.

Category	Drugs	Object	Drug efficacy	Clinical trials/reference
Pim kinase inhibitor + IMiD	SGI‐1776 + Lenalidomide	Subcutaneous tumor model (MM1R cell)	Nearly complete inhibition after 3 weeks treatment	^36^
		Systemic tumor model (MM1R cell)	Median survival 73 days (21 days longer than DMSO)	
Pim kinase inhibitor + PI	SMI‐16a + bortezomib	KMS11 cells	SMI‐16a reduced pim‐2 upon treatment with bortezomib	^29^
		RPMI8826 cells		
	SMI‐16a + carfifilzomib	RPMI8826 cells	Enhanced cytotoxicity	
		KMS11 cells	Reduced viability	
	JP11646 + bortezomib	MM1S cells	Viability reduced from 18% to 2%	^30^
		U266 cells	Viability reduced from 85% to 53%	
Pim kinase inhibitor + small molecule inhibitor	PIM447 + BYL719	Relapsed and refractory multiple myeloma	Not available yet	NCT02144038
	INCB053914 + Itacitinib	INA‐6 xenografts model	Synergistic inhibition of tumor	^27^
			Combination Index = 0.720	
			95% CI: [0.256, 0.924]	
	(Z)‐5‐(4‐propoxybenzylidene) thiazolidine‐2,4‐dione + LY294002	INA‐6 cells	Number and viability significant decline	^79^
Pim kinase inhibitor + dexamethasome	PIM447 + dexamethasome	MM1S cells	Highly synergistic range	^4^
		RPMI8826 cells	Combination Index	
			(CI) = 0.096	
	JP11646 + dexamethasome	MM1S cells	Viability reduced from 55% to 8%	^30^
Pim kinase inhibitor + IMiD + PI (Pim‐Pd)	PIM447 + bortezomib + dexamethasome	RPMI8826 cells	Combination Index, CI = 0.002	^4^
	PIM447 + lenalidomide + dexamethasone		Combination Index, CI = 0.065	
	PIM447 + pomalidomide + dexamethasone		Combination Index, CI = 0.077	

#### 5.2.1 Double drugs combination

##### Pim kinase inhibitor with IMiDs

The IMiDs currently used in clinical practice of MM management are lenalidomide, pomalidomide, and thalidomide. which are also among the mainstays in the management of MM.^87^ Lenalidomide is the most utilized in patients in relapsed settings and maintenance medical care after autologous stem cell transplantation.^88^ In MM cell lines and myeloma xenograft models, pan‐pim kinase inhibitors provide additional CRBN binding sites for IMiD, thus enhancing their role, and upregulating recruitment and ubiquitination. In addition, more effective degradation of IKZF1 and IKZF3^54^ and their downstream substances: IRF4 and c‐Myc were achieved. Nearly total inhibition of myeloma cells was observed in the subcutaneous xenograft mouse model with the combined therapy of SGI1776 and lenalidomide after 3 weeks of treatment. Moreover, in the systemic myeloma model, the group that received the combination survived an average of 3 weeks longer than the control group.^89^

##### Pim kinase inhibitor with PI

Pim‐2 can be degraded by proteasomes rather than ubiquitination.^90^ Bortezomib and carfilzomib are two PIs that can increase pim‐2 kinase levels without increasing the level of pim‐2 mRNA in MM cells. However, similar results were not found for the IMiDs, lenelidomide and pomalidomide.^75^

SMI‐16a could still downregulate pim‐2 kinase in KMS11 and RPMI8226 cells. Bortezomib, an NF‐κB pathway inhibitor, when combined with SMI‐16a,^75^ attenuates the side effects caused by bortezomib alone.^91^ A combination of SMI‐16a and carfilzomib increased the toxicity of RPMI8226 cells and reduced the viability of KMS11 cells.^75^ Combined with JP11646 and bortezomib, cell viability decreased from 18% (single‐drug therapy) to 2% in MM1.S, whereas in U266, a more resistant cell line, the cell viability decreased from 85% to 53%.^74^ This suggests that the enhanced cytotoxic activity of PIs by pim kinase inhibitors could be associated with the suppression of pim‐2 effects.

##### Pim kinase inhibitor with other small molecule inhibitors

LY294002 is not only an inhibitor of the PI3K/Akt pathway,^92^ but also acts a participant in the IGF‐1‐induced signaling pathway. The expression of pim‐2 upregulated by BMSCs was not affected by LY294002 in MM cells. However, LY294002 in combination with (Z)‐5‐(4‐propoxyphenylmethylene)thiazolidine‐2,4‐dione, a pim kinase inhibitor, reduced the number of INA‐6 cells irrespective of the presence of BMSCs.^30^ Compound 16 (GDC‐0339) with a diaminopyrazole was identified as a pan‐pim inhibitor that had high oral bioavailability and was well tolerated in safety trials. According to the study, compound 16 achieved a greater anti‐myeloma effect by lowering its IC50 and thus binding better to PI3 inhibitors.^93^ An increase in pim‐1 kinase can mediate tumor resistance to PI3K inhibitor therapy, which indirectly suggests the expected effect of combining pim kinase inhibitors with PI3K inhibitors.^94^ A clinical trial of LGH447 and BYL719 (a PI3K inhibitor) in relapsed/refractory MM patients is in progress (NCT02144038).^95^

Aberrant JAK2 signaling and myeloproliferative neoplasms are closely related; however, the data suggest that tumor cells are insensitive to anti‐JAK2 treatment regimens; therefore, effective treatments are needed.^96^ When INCB053914 (100 mg/kg orally BID) was combined with itacitinib (60 mg/kg orally BID), which can inhibit JAK1 or JAK1/2, potent synergistic inhibition of tumor growth was detected after 8 days in INA‐6(MM) xenografts, compared with that of each agent alone (Combination Index = 0.720 [0.256, 0.924]). The combination regimen also induced a slightly stronger decline in MYC levels than that observed with either INCB053914 or itacitinib alone. Among other anticancer agents, 19 in 65 showed variable degrees of synergistic effects with INCB053914 in KMS‐11, KMS‐12‐BM, and MM1.S cells.^76^ In addition, pim kinase inhibitors, combined with JAK2 inhibitors, overcame drug resistance and suppressed the proliferation of MPN cells.^97^

##### Pim kinase inhibitor with dexamethasone

In an in vitro experiment, a highly synergistic effect (CI = 0.096) was observed between pim447 (200 nM) and dexamethasone (10 nM) in MM1S. Similar results were observed in RPMI‐8226 cell lines.^29^ The combination of JP11646 and dexamethasone led to a decrease in the viability of MM1.S cells from 55% to 8%.^74^

#### 5.2.2 Triple drugs combination (pim‐Pd)

Pim‐Pd refers to a combination of the pim kinase inhibitor IMiD and PI. Paíno combined pim447 with lenalidomide + dexamethasone and pomalidomide + dexamethasone in RPMI‐8226 cell lines. The results showed that the combination of the three drugs led to the death of approximately all myeloma cells at low doses (pim447 at 50 nM, Poma at 100 nM, and Dema at 2.5 nM).^29^ PIM‐Pd treatment induced a cell cycle block in MM.1S and NCI‐H929 cell lines, with an increased percentage of cells in the G0‐G1 phase and a decrease in the proliferative phase (S and G2‐M). Furthermore, the survival of treated mice was prolonged by inhibiting the translation of mTORC1 and c‐Myc, which later interrupt eIF4E function.^3^

## 6 PIM KINASE AND IMMUNE MODULATION

The survival of tumor cells is closely related to the immune microenvironment of tumor tissues.^98^ However, how pim kinase regulates immune cells and the immune microenvironment, as well as the roles of pim kinase inhibitors in MM patients' immune modulation, remain unclear.

### 6.1 T cells

β‐selectin is one of the earliest management points in the process by which immature naive thymocytes differentiate, mature, and expand the required c‐Myb protein downstream of pim‐1.^99^ The study by Leduc et al. found that overexpression of pim‐1 in two independent knockout mouse models resulted in massive proliferation of CD4(+)CD8(+) double‐positive thymocytes.^100^ IL‐9/IL‐9R signaling is important for the regulation of T‐cell alloresponses by pim‐2 kinase. A variety of molecules, including JAK1, STAT1, IFN‐γ, IRF7, and TNF‐α, which cause differences in the signaling and survival of T cells, are upregulated in T cells without pim‐2.^48^ Pim‐2‐deficient T cells have a greater capacity for survival, proliferation, and pro‐inflammatory cytokine production, primarily owing to the downregulation of SOCS‐1 and p73 and upregulation of IL‐9R.^101^ Elevated levels of molecules involved in the antitumor response, such as FASL, HDAC9, TBX21, TNF‐α, IFN‐γ, etc. are observed as well. However, the function of pim‐1 and pim‐3 is exactly the opposite.^101^ Maintenance of CD8^+^ T cell memory requires both high levels of Eomesa expression and a continuum of intracellular NF‐κB signaling. pim‐1 kinase supports memory fitness in CD8^+^ T cells by supporting the expression of Eomes, and thus the memory of CD8^+^ T cells.^102^ Pim kinase is expressed more in Th1 than Th2 cells,^103^ whereas pim‐1 was reported to be strongly upregulated in CD4^+^ cells from human cord blood differentiated toward Th1 cells, but not in cells toward Th2 cells.^104^ However, pim‐2 kinase can phosphorylate Foxp3, which leads to reduced suppressive functions of Treg cells.^105^ Similarly, the suppressive function of Treg cells can be enhanced by pharmacological inhibition of pim‐2 kinase or its knockout.^105^

### 6.2 B cells

Pim‐1 deficiency causes a marked reduction in IgM (−) B‐cell precursors.^106^ Transduced pre‐B cells exhibit substantial proliferation with the co‐expression of pim‐1 and Myc in vitro, which is IL‐7 independent while preventing their differentiation into IgM (+) immature cells.^107^ Unlike pim‐1, pim‐2 prevents the transition of pre‐B cells during the G1/S phase and DNA breaks may be repaired erroneously.^108^ In plasma cells, IL‐6 can trigger the expression of pim‐2, resulting in a pim‐2‐dependent anti‐apoptotic effect on malignant plasma cells. This also implies a role for pim‐2 in the malignant proliferation of myeloma cells.^109^ Stimulated with BLyS (B‐lymphocyte stimulator) pim‐2‐deficient B cells were protected from death, but this protection was completely nullified when treated with rapamycin (an mTOR inhibitor).^110^ Transformation of pre‐B cells requires pim‐1 and pim‐2 kinases, which are involved in the modification and regulation of SOCS‐1.^111^

### 6.3 NK cells

IL‐12 and IFN‐α (two Th1‐specific cytokines) temporarily upregulate the mRNA levels of pim‐1 and pim‐2 in NK cells from the human peripheral blood.^104^ Treatment with ssRNA‐pim‐3‐shRNA bifunctional vectors enhanced the activation of NK cells while reducing the ratio of intratumoral Tregs to MDSCs.^112^

Immunomodulation is an important issue in MM research.^60^ However, reports of pim kinase in the immune system of MM patients are still rare, and further research is needed to explore how pim kinase affects the mechanism of action of multiple immune cells and immune factors in MM.

## 7 CONCLUSION

Pim kinase plays a vital role in cellular pathways, including JAK/STAT, c‐Myc, and BAD/Bcl‐2, and acts as a promoter of cell proliferation by phosphorylating several molecules including mTORC1, 4EBP1, and BAD. All these factors have been proven to be important in tumor cell growth and development. Recently, pim kinases have also been shown to regulate the cell cycle, for example, by activating CDC25A/CDC25C and changing cyclin‐dependent kinases such as CDK2, CDK4, and CDK6. Studies on pim kinase in MM bone disease have shown that pim kinase interacts with OCs and OBs via the NF‐κB and RANKL pathways, increasing osteoclastic activity and suppressing osteoblastic differentiation, which causes devastating bone destruction and rapid loss of bone. Pim kinase also regulates the differentiation and proliferation of lymphatic B cells and T cells and NK cells via activation of the NF‐κB, MYC, and mTOR pathways. All these effects of pim kinase indicate pim kinase as a comprehensive therapy target in MM.

With more pim kinase inhibitors being discovered, the therapeutic options for patients with MM are increasing. All current myeloma drugs have certain side effects, in the case of pim, this may manifest as an increase in pim kinase levels. Based on the available experimental findings, we can say that this side effect can be largely reduced or even eliminated if pim kinase inhibitors are used in combination.

## 8 FUTURE PROSPECTS

The pim kinase family was only discovered and defined in the 1990s, but its role in tumors, particularly hematologic tumors, had not received attention until 2010. Nevertheless, the role of pim kinases cannot be underestimated, as evidenced by the fact that a number of pim kinase inhibitors have entered phase III clinical trials in a short time. However, this emerging field still has many unknowns to explore.

Current studies have failed to delve into and elucidate the role of pim kinase in the clonal proliferation of tumor cells, for example, there are two opposing views on the regulation of the cell cycle in myeloma cells. Tumor cells generally obtain energy rapidly through anaerobic glycolysis, as do myeloma cells. If pim kinase is able to regulate myeloma cell metabolism then it has the opportunity to influence all cellular activities and even the production of monoclonal immunoglobulins. On the other hand, the involvement of regulatory factors in the transcriptional and translational processes of pim kinases in myeloma cells is an area for future research. The above is focused on the tumor itself, but as the second most common hematologic tumor, myeloma lesion is in the patient's bone marrow, so myeloma inherently has an immune microenvironment containing a large number of naïve immune cells. It has been shown that myeloma cells can secrete cytokines to alter the bone marrow microenvironment to suit themselves. Whether pim kinase, which has been shown to regulate the proliferation and differentiation of immune cells, can regulate the immune microenvironment of myeloma remains to be investigated.

Although single‐agent clinical trials such as pim447 have been beneficial for patients with MM, more clinical data are urgently needed. Bone disease is an important complication of myeloma, and osteolytic bone destruction is also involved in determining disease staging. We know that pim kinase is able to modulate osteogenesis and osteolysis, However, the current clinical trials were conducted in patients with relapsed/refractory MM and did not provide a good view of the effect of inhibitors on bone disease progression. Conducting trials in patients with newly diagnosed MM while tracking patients' blood calcium and bone destruction will help determine the efficacy of the drug. Combining pim kinase inhibitors with existing myeloma therapeutic agents to assess treatment efficacy and the occurrence of side effects will provide a reliable basis for the integrated management of myeloma patients in the future.

## AUTHOR CONTRIBUTIONS

**Zhaoyun Liu:** Methodology (equal). **Yunhe Zhang:** Conceptualization (equal). **Yixuan Guo:** Investigation (equal). **Hao Wang:** Investigation (equal). **Rong Fu:** Project administration (equal).

## CONFLICT OF INTEREST STATEMENT

None.

## Supporting information


**Figure S1.** Supporting information

## Data Availability

Data sharing is not applicable to this article as no new data were created or analyzed in this study.
