# Supplementary material for: An overview of pim kinase as a target in multiple myeloma
Source: Cancer Med. 2023 May 10;12(10):11746–59. doi: 10.1002/cam4.5797 (PMC10242321; doi:10.1002/cam4.5797)
Supplement: Supplementary file 1 — Figure S1. Supporting information [file CAM4-12-11746-s001.pptx]

## Slide 1
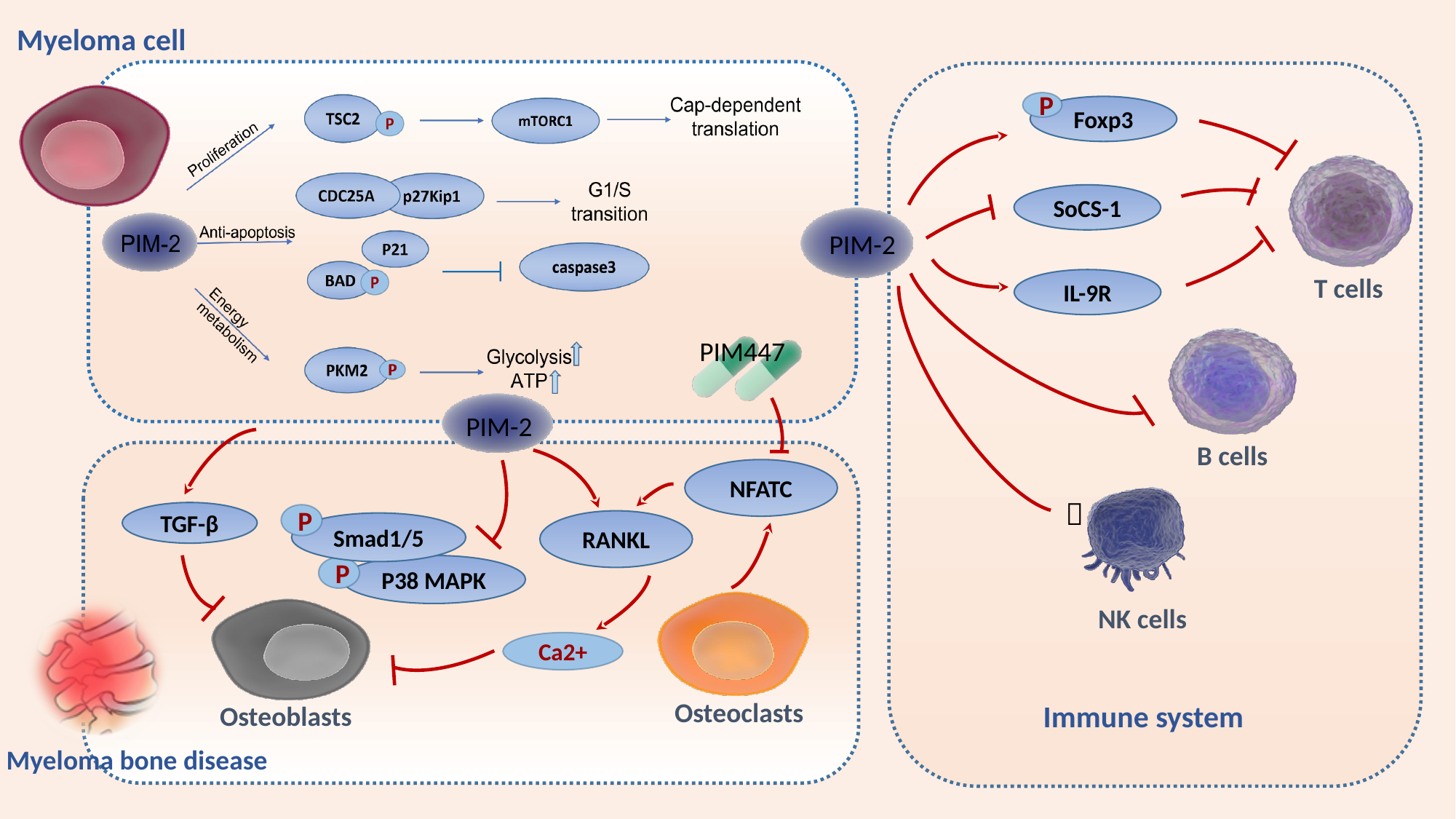

Myeloma cell
PIM-2
PIM-2
P
Foxp3
SoCS-1
T cells
IL-9R
PIM447
B cells
NFATC
？
TGF-β
P
Smad1/5
RANKL
P38 MAPK
P
NK cells
Ca2+
Osteoclasts
Immune system
Osteoblasts
Myeloma bone disease
